# Emerging role of gut microbiota extracellular vesicle in neurodegenerative disorders and insights on their therapeutic management

**DOI:** 10.1002/imo2.33

**Published:** 2024-10-25

**Authors:** Adil Hassan, Yin Huang, Mahjabina, Otmilda Hapsari Sudiro, Xiangxiu Wang, Jingqin He, Yan Wei, Xiaoling Liao, Guixue Wang

**Affiliations:** ^1^ Department of Materials Science, college of Materials and New Energy, Chongqing Key Laboratory of Nano/Micro Composite Materials and Devices Chongqing University of Science and Technology Chongqing China; ^2^ JinFeng Laboratory Chongqing China; ^3^ Department of Bioengining, College of Bioengineering, Key Laboratory for Biorheological Science and Technology of Ministry of Education, State and Local Joint Engineering Laboratory for Vascular Implants, Bioengineering College Chongqing University Chongqing China; ^4^ Department of Gastrointestinal Surgery The First Affiliated Hospital of Chongqing Medical University Chongqing China; ^5^ Institute of Cardiovascular Research, Key Laboratory of Medical Electrophysiology, Ministry of Education & Medical Electrophysiological Key Laboratory of Sichuan Province Southwest Medical University Luzhou Sichuan China

**Keywords:** brain dysfunction, drug delivery system, gut microbiota extracellular vesicle, neurodegenerative disorders, neuro‐inflammation

## Abstract

Targeting gut flora to lower sickness risk is a growing scientific subject. The intricate network of microorganisms (gut microbiota) in the human intestines regulates many physiological systems and may be important for general health. Recent research has shown a dynamic relationship between gut microbiota and central nervous system (CNS). Dysbiosis is key to establishing and progressing human diseases, including neurodegenerative disorders. Recently, gut microbiota extracellular vesicles (GMEVs) have been suggested as brain‐gut communication carriers. Vesicle components contact immune receptors, initiating neuroinflammatory immune responses and causing neurodegenerative diseases. This study seeks to explain how the gut microbiota and its extracellular vesicles cause or worsen neurodegenerative diseases. We also highlighted recent advances in our understanding of these GMEVs' and cargo's routes, which could be used in drug delivery treatments. This study also examines the current state and potential therapeutic effects of GMEVs on neurodegenerative illnesses.

## Highlights

- Dysbiosis, which refers to abnormalities in the bacteria that live in the gut, may have a vital role in the development and progression of neurodegeneration disorders.
- Gut microbiota extracellular vesicles have the potential to function as messengers, transmitting messages between the gut and the brain.
- Gut microbiota extracellular vesicles can worsen neurodegenerative conditions but also hold potential as drug delivery systems for innovative treatments.

## Graphical abstract

Gut microbiota extracellular vesicles play (GMEVs) a vital role in maintaining the health of the digestive system and facilitating proper brain functioning. Gut dysbiosis exacerbates gut inflammation, facilitating the entry of harmful substances into the bloodstream. Systemic inflammation has the potential to impair the integrity of the blood‐brain barrier. Neuroinflammation and neurodegenerative illnesses can be triggered by the infiltration of inflammatory substances into the brain. A healthy microbiota safeguards the integrity of the intestinal barrier and inhibits the proliferation of harmful pathogens. In addition, GMEVs convey beneficial chemicals and signaling molecules, enhancing intestinal health and reducing systemic inflammation. An intact gut microbiota provides protection against inflammation and supplies essential nutrients and signaling molecules for optimal brain function, therefore playing a crucial role in maintaining general health. Notes: ↑ means increase, ↓ means decrease.

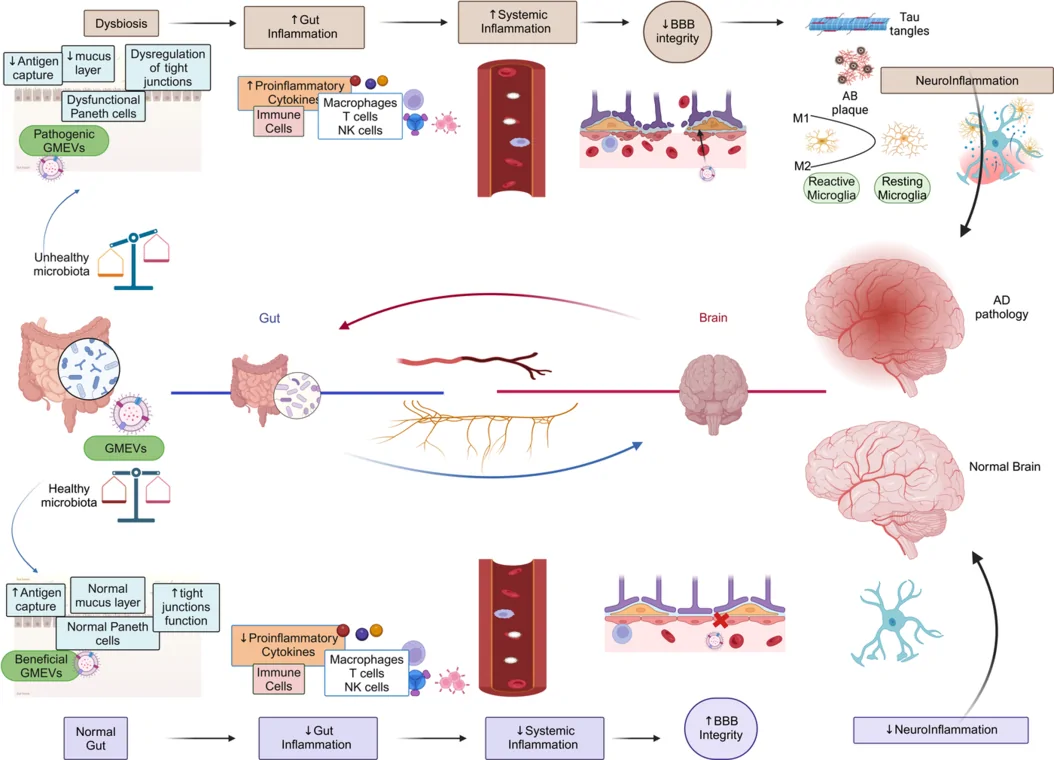

## 1 INTRODUCTION

Neurodegenerative disorders (NDs) pose a substantial and increasing threat to worldwide public health and impact millions of people globally [1]. Degeneration and death of neurons are the root causes of neurodegenerative illnesses, which in turn produce substantial impairments in mental, cognitive, and physical functions [2]. Several NDs are quite prevalent, including Alzheimer's disease (AD), Parkinson's disease (PD), amyotrophic lateral sclerosis (ALS), and Huntington's disease (HD) [3]. The diagnosis and treatment of NDs are challenging to accomplish due to the complicated progression of these disorders, which involves a wide range of factors, such as heredity, environmental exposure, and the natural process of aging [4, 5]. In addition, mitochondrial dysfunction, misfolded proteins, neuro‐inflammation, and oxidative stress (OS) are frequently observed in patients who suffer from NDs. Additionally, there has been a commensurate rise in the incidence of NDs in tandem with the global increase in the population that is getting older. Consequently, there is an urgent need for the development of innovative treatments to manage these illnesses properly [6].

The gut microbiota (GM) is a diverse and complex community of microorganisms that inhabit the gastrointestinal (GI) tract, predominantly in the large intestine. With a population exceeding 100 trillion, these bacteria play a vital function in maintaining stability and balance in the human body [7, 8, 9]. The complex microbial community exerts significant influence on several biological processes, including the regulation of the immune system, the metabolism of food, and even the psychological state of the individual [10]. Emerging research suggests that an alteration in the composition of the GM may contribute to the advancement and onset of various diseases, including AD, PD, HD, and ALS [11]. The GM is regarded as an essential part that has the potential to influence brain function through several different channels. These channels include the immunological pathway, the vagus nerve, the circulatory system, and the hypothalamic‐pituitary‐adrenal axis, as well as the lymphatic and glymphatic circulation [11, 12]. Recent studies have extensively explored the important connection between the gut‐brain axis (GBA) and NDs. This research has provided growing data indicating a possible association between GM and the onset of NDs. Studies have shown that an imbalance in the GM associated with AD can be passed on, potentially leading to a loss in cognitive function. This transmission is associated with an elevated level of Tau phosphorylation, which is a characteristic feature of AD. Potential therapeutic approaches for addressing cognitive impairment associated with AD include modulating the GM with probiotic therapies and targeting the butyric acid‐GSK3β pathway [13, 14, 15, 16, 17]. In light of this, changes in the composition and diversity of the microbiota in the gut not only cause disorders related to the gut, but also contribute to diseases that affect the GI tract and NDs [18]. The gut microbiota extracellular vesicles (GMEVs) have recently become a subject of intense interest in host‐microbiota communication [19]. Different biomolecules can be transported across biological barriers by extracellular vesicles (EVs), which are membrane‐enclosed particles that are secreted by almost all cell types [20]. Despite significant research projects, the causes and development of several NDs still need to be fully comprehended, highlighting the need for a new understanding of the fundamental processes involved. Recent research has highlighted increasing evidence that the microbiota and their secreted EVs play a role in developing and advancing NDs [21]. GMEVs can trigger specific signaling in the brain, potentially by directly crossing the blood‐brain barrier (BBB), these vesicles have different effects on various brain cells, including neurons, astrocytes, and microglia, due to their rich and diverse cargo of proteins and small ribonucleic acids (RNA) [22, 23]. The GMEVs have demonstrated the ability to control the expression of genes in the brain and trigger the development of illnesses at different phases of neuro‐inflammation and neurodegeneration. As a result, they play a direct role in the onset of numerous conditions, including stroke, AD, PD, and dementia [3, 24, 25, 26]. On the other hand, GMEVs obtained from specific types of bacteria, specifically those found in the gut and known for their probiotic qualities, have recently been demonstrated to have specific therapeutic benefits for different neurological illnesses. Therefore, GMEVs can both contribute to and treat neuropathological problems [26, 27, 28].

This study aims to investigate the function and pathways of GMEVs in NDs. We will examine the existing literature on the role of GMEVs on NDs. This review will focus on experimental results from animal models and human research. In addition, we will explore the various mechanisms, such as neuro‐inflammation, OS, mitochondrial dysfunction, protein misfolding, and aggregation, and other pathogenic processes linked to neurodegeneration. Lastly, we will examine and discuss recent advances in our knowledge of the many pathways taken by these GMEVs and their cargo, which are potentially valuable for the future delivery of drug therapies, emphasizing future research paths in this dynamically emerging area.

## 2 OVERVIEW OF GM AND EVs

### 2.1 Gut microbiota

The collection of microbes found in the human digestive tract is commonly known as the gut microbiome [29]. Previous studies have highlighted the astonishing richness of the GM, which consists of over 100 trillion cells, mostly bacteria, fungi, archaea, and viruses [30]. The GM is responsible for a wide range of functions, including enhancing nutritional absorption and extracting energy. In addition, it adds distinctive metabolic pathways and metabolites necessary for the proper functioning of the body [8]. The GM also acts as a natural defense system, encouraging the growth of natural and acquired immunity and blocking the passage of potentially harmful organisms that might upset its fragile balance [31]. Besides its metabolic and defensive roles, the microbial community also directly influences the composition of the GI. It controls the functioning of the intestinal cells, the inter connections between these cells, and mucus production. This contributes to the overall well‐being and equilibrium of the GI system [18]. GM imbalance, often called “dysbiosis,” refers to a disruption or imbalance in the composition and activity of the intestinal flora. This disruption is characterized by changes in the variety of microorganisms, abundance, and metabolic activity [32]. Several health problems have been associated with dysbiosis, with particular emphasis on immunological instability and intense inflammation, such as inflammatory bowel disease, metabolic syndrome, cardiovascular disease, and neuropsychiatric illnesses [33, 34].

Recent scientific developments have widened our knowledge of gut flora by uncovering the complex network of interactions mediated by GMEVs [35]. GMEVs, such as exosomes and outer membrane vesicles (OMVs), serve as intercellular communication vehicles, allowing gut bacteria and host cells to communicate and exchange compounds and genetic material [36, 37]. This bidirectional communication system is involved in a range of physiological activities, such as immunological modulation, metabolic regulation, and neurobehavioral functions [38, 39].

Recent data indicates a significant connection between changes in GMEVs caused by dysbiosis and the development of NDs [40]. The alterations in the content of EVs caused by dysbiosis, such as microRNAs (miRNAs), lipids, and proteins, can potentially affect neuro‐inflammation, the integrity of the BBB, and the health of neurons [41, 42]. Moreover, chemicals produced by GM and transferred by GMEVs might influence neurodegenerative pathways, namely those related to protein misfolding and aggregation, which are associated with illnesses such as AD and PD [43, 44].

**Figure 1:**
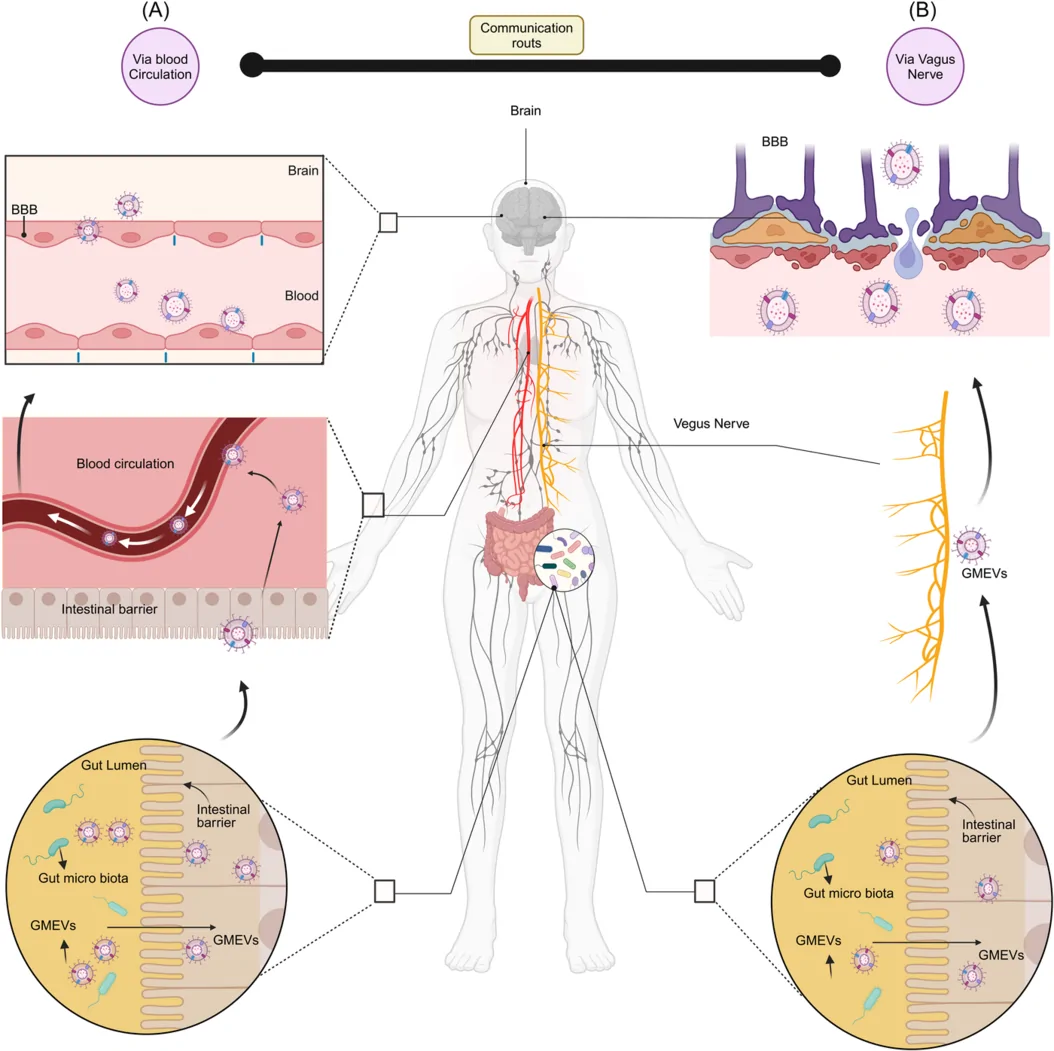
Gut and brain communication via gut microbiota extracellular vesicles. (A) These nanovesicles traverse our intestinal walls and enter our blood circulation. Subsequently, they may traverse into the brain and impact cerebral cells. (B) They also use the vagus nerves as a means of transportation to reach the brain.

The GBA is a two‐way network that connects the intestinal normal flora, GMEVs, and the CNS. It plays an essential part in controlling normal and abnormal biological processes, such as NDs [45]. The GM, which consists of a wide range of microbial communities, interacts with the host through many processes, such as the creation of bioactive compounds and GMEVs [46]. These GMEVs, carrying miRNAs, proteins, and other biologically active substances, operate as intermediaries, enabling communication between GM and the CNS [46, 47]. On the other hand, the CNS can alter the composition and function of the GM through the autonomic nervous system and neuroendocrine pathways, which in turn affects the proliferation of microorganisms, their metabolism, and the release of GMEVs [41, 48]. Changes in CNS signaling, which are hallmarks of NDs, can impair this bidirectional connection, which in turn exacerbates an imbalance and neurological inflammation [49, 50]. The bidirectional communication between the gut and brain is shown in Figure 1.

### 2.2 Gut microbiota extracellular vesicles

EVs have become increasingly recognized as essential mediators of communication across different kingdoms, such as the GM and mammals [51, 52]. EVs are produced by all domains of life, including eukaryotes, bacteria, and archaea. These particles can influence cell function either within the same cell (autocrine) or in nearby cells (paracrine) [51, 53, 54]. Cellular communication occurs through the release of EVs, which are particles enclosed by a membrane [55, 56]. GMEVs possess the capacity to modify biological activities and influence physiological processes as a result of the bioactive cargo they carry. The cargo comprises proteins, lipids, nucleic acids, and small molecules (metabolites) [57, 58, 59]. The existence of bacterial extracellular vesicle (BEVs) was initially documented in *Escherichia coli* during the 1960s [60, 61, 62], while Bryant, W.A. proposed in 2017 that GMEVs may play a role in the proliferation of the GI tract [63].

**Figure 2:**
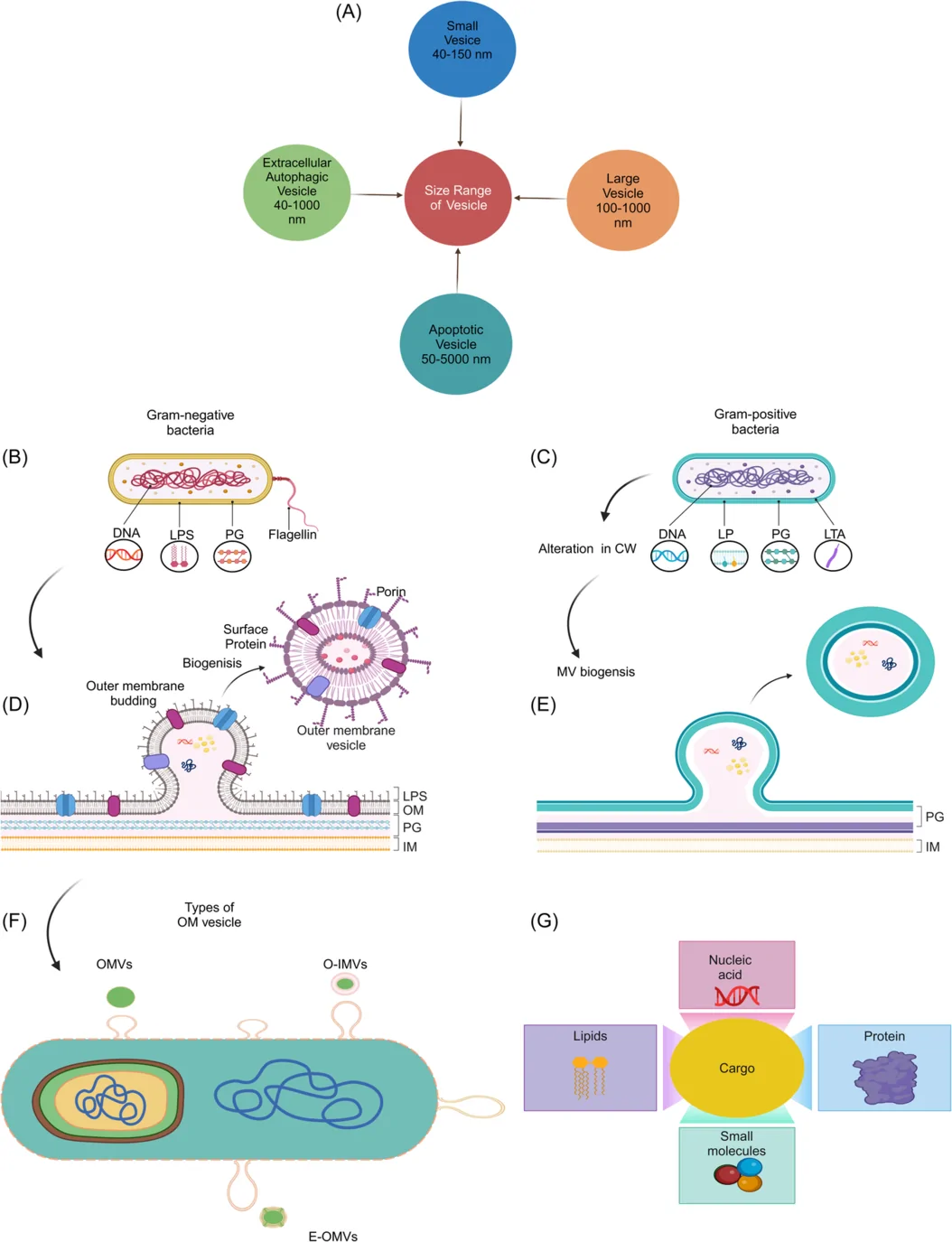
Gut microbiota extracellular vesicles and its biogenesis. Presents a comprehensive depiction of (A) different size ranges of EVs, (B, C) Bacteria structure, (D, E) their procedure of biogenesis, (F) the various classifications of outer membrane vesicles and (G) their cargo. Gut microbiota extracellular vesicles of gram‐negative bacteria (GMEVs) are produced either by the process of blebbing, when the outer membrane forms little protrusions, or during explosive cell lysis. The formation of typical outer membrane vesicles (OMVs) results from the protrusion of hydrophobic molecules or the instability of peptidoglycan production, leading to outer membrane blebbing. The peptidoglycan layer's weakness causes the inner membrane to bulge out, resulting in the formation of explosive outer membrane vesicles or outer‐inner membrane vesicles. GMEVs of gram‐positive bacteria are produced by a process called bubbling cell death. During this process, an enzyme called endolysin breaks down the peptidoglycan layer, leading to cell lysis and the production of cell membrane vesicles. EV, extracellular vesicle.

Mammals categorize EVs primarily into exosomes, microvesicles, and apoptotic bodies based on their biosynthesis and size. Exosomes are EVs derived by endocytosis with a size range of 30–150 nm. In contrast, microvesicles have a diameter that falls within the 100–1000 nm range and are created from the plasma membrane. On the other hand, apoptotic bodies have a size ranging from 50 to 5000 nm and are produced by cells undergoing apoptosis [64]. Plants are capable of releasing EVs that have biogenesis, content, and appearance similar to exosomes seen in mammals. These plant EVs are sometimes referred to as “exosome‐like nanovesicles” [65]. According to Cuesta et al. [66] GMEVs are similar to EVs found in eukaryotic organisms. These similarities include their size range of 10–400 nm, spherical shape, and physicochemical properties, such as their stability when frozen or at a temperature of 37°C. Nevertheless, several distinctions have been identified regarding attributes such as arrangement, resistance to elevated temperatures, constitution, and their origin [66]. A notable similarity between eukaryotic and GMEVs is their capacity to carry genetic material, specifically miRNAs or miRNA‐like molecules. These molecules have been detected in various types of EVs, including mammalian exosomes, microvesicles, apoptotic bodies, plant exosome‐like nanovesicles, and bacterial EVs [67, 68, 69]. Different sizes of EVs are shown in Figure 2A.

### 2.3 Biogenesis of bacteria extracellular vesicles

BEVs are membrane‐bound structures that have significant functions in diverse biological processes. BEVs exhibit distinct characteristics compared to eukaryotic organisms, including their formation process, content, and size [70]. Gram‐negative bacteria are renowned for their capacity to generate OMVs, whilst Gram‐positive bacteria employ distinct, less comprehended mechanisms for generating EVs. Gram‐negative bacteria not only make OMVs, but also generate outer‐inner membrane vesicles and explosive OMVs. These vesicles have different biogenesis pathways and cargo profiles [71, 72, 73, 74, 75, 76]. In 1990, Gram‐positive bacteria were found to produce EVs, defying the long‐held belief that their influential peptidoglycan structures prevented them from vesicle production [75, 77]. Gram‐positive EVs lack lipopolysaccharides (LPS) but contain peptidoglycan and proteins [54, 77, 78, 79]. The mechanics of GMEVs release remain unknown, however, turgor pressure may drive GMEVs through cell wall pores and related enzymes may degrade peptidoglycan barriers [54]. GMEVs from *Staphylococcus aureus* contained peptidoglycan‐degrading enzymes, supporting the latter concept. Toyofuku et al. [77] found that prophage‐encoded endolysin enzymes in *Bacillus subtilis* strains hydrolyze peptidoglycan to aid GMEVs biogenesis. Additionally, *S. aureus* enzymes such as phenol‐soluble modulins and autolysins contribute to GMEVs production [77, 80, 81, 82]. The GMEVs and their biogenesis are shown in Figure 2B–F.

### 2.4 GMEVs molecules cargo

GMEVs serve as versatile carriers for a diverse repertoire of biomolecules, including proteins, nucleic acids, small molecules, and lipids. These cargos are meticulously packaged within the GMEV membrane, facilitating their delivery to target cells.

#### 2.4.1 GMEVS and their protein cargo

GMEVs from GM contain many proteins that aid host‐microbe interactions. These proteins are essential for host physiology and immunological responses [41]. Gram‐positive bacteria of the gut release GMEVs with membrane and cytoplasmic proteins that work in many cell functions. Pathogenic strains carry disease‐causing chemicals via GMEVs [80, 83, 84, 85]. Gram‐negative bacteria create GMEVs that contain a high concentration of periplasmic and outer membrane proteins [86, 87]. These proteins play a crucial role in bacterial development, establishment, persistence, and evading the immune system [88, 89]. GMEVs proteins can regulate dendritic cell maturation and cytokine production, which can influence immunological responses [90].

#### 2.4.2 GMEVS and their nucleic acid cargo

GMEVs contain DNA, RNA, and short non‐coding RNAs. These vesicles are essential for bacterial genetic exchange and affect host gene expression and immunological responses [91]. Microbial extracellular RNA (exRNA) affects epithelial and immunological cell inflammatory cytokine production [92, 93]. Microbial exRNA may also regulate epigenetics like eukaryotic long non‐coding RNAs [94]. New findings indicate that the genetic material carried by GMEVs might have implications for the development of NDs. The presence of nucleic acids within GMEVs underscores the effectiveness of vesicle‐mediated delivery systems. Unlike free nucleic acids, which are prone to degradation, GMEVs safeguard their cargo, allowing for successful transport across physiological barriers [95]. Thus, the presence of RNA in GMEVs demonstrates the strength of vesicle‐mediated delivery mechanisms, emphasizing their effectiveness in protecting and transporting biologically active substances through physiological barriers.

#### 2.4.3 GMEVs and their small molecules cargo (metabolites etc.)

The GM has a vital function in the synthesis of different metabolites, which are tiny chemicals that have a substantial impact on the physiology and well‐being of the host. Microbial metabolites function as signaling molecules that enable communication between the GM and the host, influencing several biological processes such as metabolism, immunological function, and mental health [96, 97, 98, 99, 100, 101]. Various small molecules generated from microbial metabolism comprise the metabolic component of GMEVs payload [102]. Short‐chain fatty acids, bile acids, neurotransmitters, and secondary metabolites are among the various metabolites associated with GMEVs. Intestinal flora has been found to make neurotransmitters like dopamine, norepinephrine, and γ‐aminobutyric acid (GABA) [103]. Metabolomic profiling has recently undergone significant development, enabling a thorough examination of metabolites associated with GMEVs and understanding their essential roles in interactions between hosts and microbes [104]. Metabolites associated with GMEVs have been found to impact the host's physiology and susceptibility to diseases. The short‐chain fatty acids produced by GM can play a crucial role in regulating the host's immune responses and inflammatory signaling pathways, thus contributing to the maintenance of gut homeostasis [105].

Hence, it is crucial to understand the structure and importance of metabolites associated with GMEVs. This knowledge is essential for uncovering the complex mechanisms that govern the interactions between GM and the host and the effects on human health.

#### 2.4.4 GMEVS and their lipid cargo

Lipids have a vital function as structural elements of Gram‐negative bacteria OMVs. The lipids in OMVs are comparable to those in the OM of bacteria [106, 107]. However, specific distinct lipids are uniquely found in OMVs [108]. Given that entero toxigenic *Escherichia coli* OMVs contain glycerophospholipids, phosphatidylglycerol, phosphatidyl‐ethanolamine, and cardiolipin as the primary lipid components. These lipids have a role in shaping the rounded shape of OMVs [109]. Moreover, it has been found that OMVs derived from *P. syringae* contain phosphatidyl‐glycerol and phosphatidyl‐ethanolamine as the primary lipid constituents [106]. Phosphatidyl‐glycerols are commonly found in OMVs in many studies, whereas phosphatidyl‐ethanolamines are the predominant components in the outer membrane [110].

Lipid diacyl A, a constituent of LPS located in the outer membrane of Gram‐negative bacteria, is also found in OMVs [111]. *Pseudomonas aeruginosa* OMVs contains a higher concentration of negatively charged B‐band LPS compared to the neutral A‐band LPS. This is probably because of the repulsive forces between neighboring B‐band molecules in the outer membrane [112]. Conversely, OMVs derived from the oral pathogen *Porphyromonas gingivalis* exhibit a higher concentration of A‐band LPS [113]. In addition, OMVs in Gram‐negative bacteria consist of phospholipids from the outer membrane and certain lipids that are exclusive to these vesicles [106, 107]. The arrangement of fatty acids influences the flexibility and stiffness of lipid membranes [114]. *P. gingivalis* OMVs contain LPS molecules with lengthy sugar chains and diacyl lipid A [113]. Similarly, *Salmonella* OMVs also accumulate diacyl lipid A [111].

The lipid composition of cell membrane vesicles in Gram‐positive bacteria differs depending on the bacterial type, which may indicate their ability to adapt and survive in different environments [115]. Vesicles of *Bacillus anthracis* and *Streptococcus pneumoniae* contain a high concentration of saturated fatty acids with carbon chains ranging from C12 to C16 [116]. Contrarily, GMEVs from *Listeria monocytogenes* possess a higher proportion of lipids containing unsaturated fatty acids in comparison to bacterial cells [115]. The GMEVs and their molecule cargo are shown in Figure 2G.

## 3 IMPACT OF GMEVS ON ND

NDs are a diverse set of conditions characterized by the gradual deterioration of nerve cells, which pose a substantial and increasing threat to the general population worldwide [1, 117]. Although extensive studies span several years, At present, there is a shortage of effective treatments for NDs, and there is no established therapy that can stop or decelerate the advancement of these conditions [118, 119]. This highlights the importance of developing novel approaches to therapy. Recent research has shown that the disruption of the GBA, which is a two‐way communication network between the GM and the CNS, plays a role in the development of NDs [120, 121]. Specifically, GMEVs have been identified as possible agents that facilitate signaling across this pathway, affecting neuroinflammatory processes, aggregation of proteins, and dysfunction of neurons [122, 123].

Studies have demonstrated that GMEVs control brain gene expression and cause pathogenesis at most neuro‐inflammation and neurodegenerative stages. As a result, they are thought to be a contributing factor in a number of disorders, including AD, PD, etc., [24, 25]. The pathogenic and therapeutic effects of GMEVs are shown in Table 1.

**Table 1:** The pathogenic and therapeutic effect of gut microbiota extracellular vesicle.

Disease	Effect	Bacteria	Reference
Alzheimer Disease	↓ Neuro‐inflammation ↑ cognitive performance	*Bifidobacterium bifidum*	[28, 124, 125, 126, 127]
		Paenalcaligenes hominis	[28]
	↑ TNF‐α Cross BBB ↑ Neuro‐inflammation	Bacteria OM	[28, 128]
Multiple sclerosis	↑ The expansion of regulatory T cells ↓ Immune autoimmune responses and inhibiting the occurrence of autoimmune	*Bacteroides fragilis*	[126, 129, 130, 131]
Parkinson Disease	↓ Neuro‐inflammation (↓ Pro‐inflammatory cytokines)	*Akkermansia muciniphila*	[132, 133, 134]
	↑ Inflammation ↑ Oxidative stress ↓ Neuronal activity ↓ Mitochondrial function	Bacteria OM	[129, 135]
Stroke	↓Neuron apoptosis ↑ MiR‐101a‐3p expression level	*Lactobacillus plantarum*	[25]

*Note*: ↑ = increase, ↓ = decrease.

Abbreviations: BBB, blood‐brain barrier; Bacteria OM, bacteria outer membrane, TNF‐α, tumor necrosis factor‐alpha.

### 3.1 Impact of GMEVs on AD

AD is the predominant kind of memory loss, marked by the buildup of amyloid‐beta plaques, neurofibrillary tangles, and neuro‐inflammation [136]. Increasing evidence indicates that GMEVs may contribute to the development of AD [24, 46]. Studies have shown changes in the content of EVs, such as amyloid‐beta peptides, inflammatory cytokines, and miRNAs, in both animal models and human patients with AD [40, 137, 138]. An imbalance in the GM, known as gut dysbiosis, may play a role in AD development. This is likely due to its ability to trigger inflammation in the brain, disrupt the protective barrier between the blood and the brain, and facilitate the spread of amyloid‐beta pathology by transferring exosomes [139, 140]. Research shows that GMEVs can induce neuro‐inflammation and compromise synaptic performance in animal models of AD [24, 139].

Recent investigation shows that GMEVs injure neurons, induce neuro‐inflammation, and affect microglia function. Teng et al. [139] observed that isoamyl amine, a normal flora metabolite, induces microglia damage, worsening age‐related cognitive decline. In addition, studies have demonstrated that GMEVs can potentially disrupt the BBB, which might allow dangerous substances and pathogens to enter the brain [28, 141].

### 3.2 Impact of GMEVs on PD

PD is a ND characterized by a steady decline in dopaminergic neurons in the substantia nigra and the accumulation of alpha‐synuclein (αSyn) protein aggregates [142]. According to recent findings, GMEVs, specifically from Gram‐negative bacteria, have the potential to contribute to PD by traveling through the lumen of the stomach and generating inflammation in the neurological system and in the local and systemic parts of the body [143]. Research on PD patients and animal models has shown changes in EVs composition. These changes include the presence of αSyn clumps, inflammatory compounds, and chemicals that facilitate the proliferation of nerve cells. The results suggest that an imbalanced distribution of gut bacteria and communication via EVs may contribute to the deterioration of neurons linked to PD. Which then may lead to inducing inflammation in the brain, disrupting the activity of mitochondria, and promoting the propagation of aberrant αSyn protein via the GBA [144, 145, 146, 147]. Furthermore, in recent years, it has been demonstrated that the utilization of mice that over‐express αSyn resulted in accumulating a more significant number of αSyn aggregates in the brains of control mice compared to those of germ‐free mice. Moreover, the oral administration of particular bacterial metabolites to germ‐free mice increased neuro‐inflammation and motor symptoms. This finding suggests that the GM and their secretions, including GMEVs may be a significant contributor to the pathology of αSyn and the activation of microglia in PD [148]. Furthermore, new research has provided insight into the possible processes by which GMEVs impact the development of PD. Studies have demonstrated that GMEVs can influence the functioning of astrocytes, which are the most prevalent glial cells in the brain. This influence results in the secretion of pro‐inflammatory cytokines and neurotoxic substances that worsen neuronal damage in PD [143, 149]. Recent findings indicate that GMEVs produced *by E. coli* could hinder the proper functioning of mitochondria in neurons, cause mitochondrial death, and trigger inflammation, resulting in heightened OS and impaired neuronal activity in PD [129].

**Figure 3:**
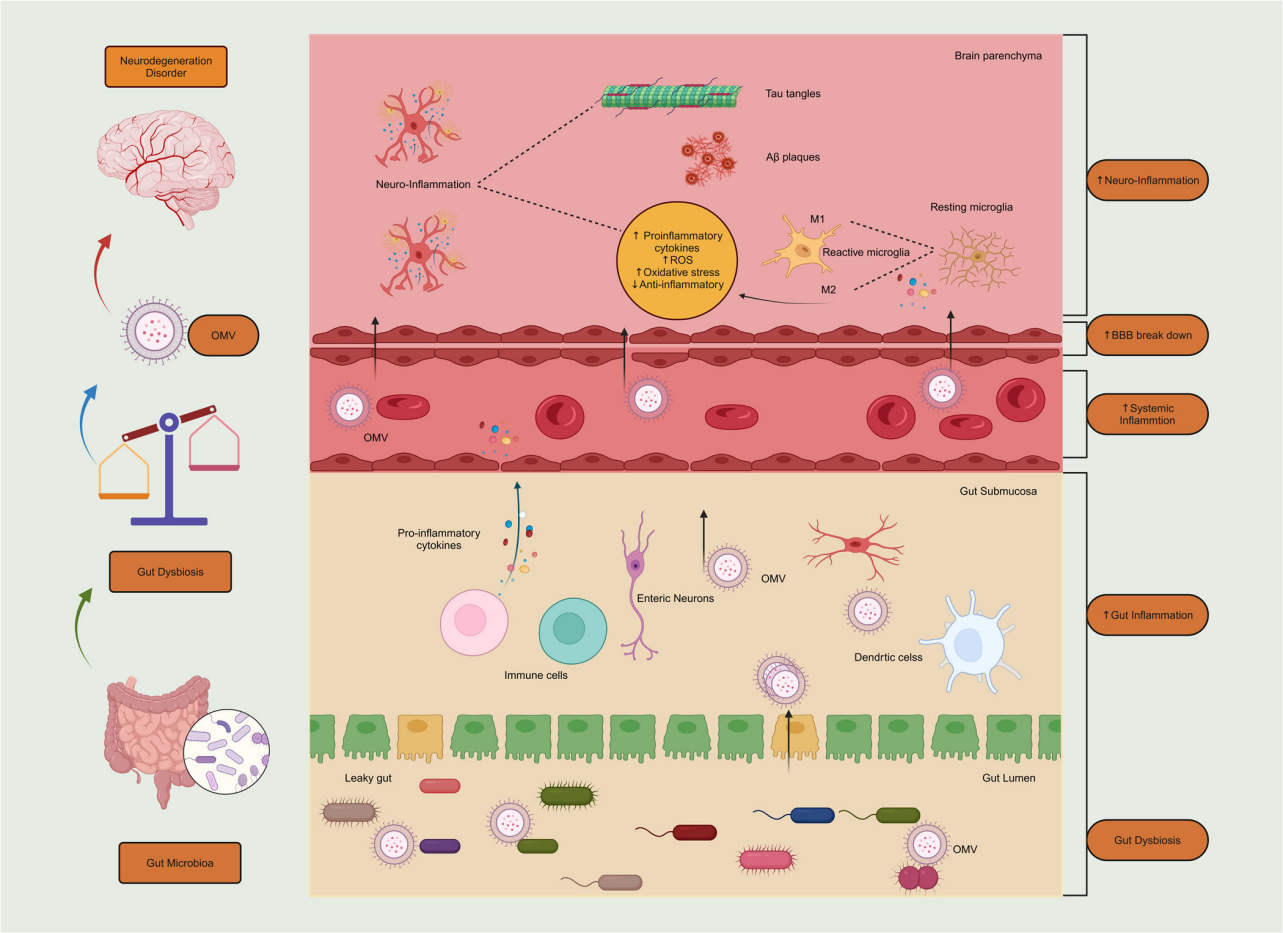
Offensive mechanism of gut microbiota extracellular vesicles on neurodegenerative disorders. This figure shows the intricate gut microbiome‐brain health relationship. Unhealthy gut microbiota releases outer membrane vesicle (OMVs) that cause gastrointestinal inflammation and gut dysbiosis. This breaks the blood‐brain barrier (BBB) and causes systemic inflammation and neuroinflammation. The cascade involves weakened epithelial barriers, autonomic nervous system‐brain connection, and central nerve system immune cell invasion. The gut inflammatory response involves enteric neurons, immunological cells, and dendritic cells in the gut submucosa. They can sense gastrointestinal changes and send brain signals via the autonomic nervous system. OMVs also pass the BBB, activate microglia, and generate pro‐inflammatory cytokines, affecting brain function and worsening neuroinflammation. *Note*: ↑ = increase, ↓ = decrease.

Moreover, research has shown that GMEVs can interfere with the enteric nervous system, sometimes called the “second brain” of the gut. This interference can result in GI dysfunction and disruption of the signaling between the gut and the brain, known as the GBA, in individuals with PD [148, 150]. Figure 3 depicts the harmful impact of GMEVs in NDs.

## 4 POTENTIAL MECHANISMS UNDERLYING GMEVS ON NDS

Exploring the complex mechanisms that explain how GMEVs affect NDs is a rapidly growing field of research that has significant implications for both fundamental scientific knowledge and medical treatment. Recent studies have provided insight into multiple functions of GMEVs in the connection between the GI tract and the brain, suggesting their involvement in developing NDs like AD, PD, etc. However, the mechanisms by which GMEVs enter the brain are not fully understood; this section examines the pathways in which GMEVs may cause brain injury, leading to NDs. Current in vivo and in vitro investigations indicate that there may be multiple mechanisms involved in the effects of GMEVs on NDs.

### 4.1 Link between GMEVs and neuro‐inflammation and OS in NDs

Neuro‐inflammation is a process linked to developing several NDs and substantially impacts the initiation and advancement of NDs such as AD and PD, etc., Multiple factors, such as trauma, infection, oxidative agents, redox iron, tau oligomers, and amyloid β‐protein (Aβ), seem to initiate neuro‐inflammation, which then leads to an abnormal release of pro‐inflammatory cytokines [151, 152]. On the other hand, OS is an imbalance in the synthesis of reactive oxygen species (ROS) and the capacity of the biological system to eliminate the reactive intermediates or reverse the damage that causes OS. An overabundance of ROS in NDs damages cells, especially in highly vulnerable neurons with rapid oxygen demand and inadequate anti‐oxidative ability [153]. OS initiates lipid peroxidation, protein oxidation, and DNA damage, which have a substantial role in developing NDs [154]. According to Cuesta et al. [66] GMEVs are essential to neuropathology as they affect brain dysfunctions by regulating the immune response. Study investigated the role of EVs harboring ROS in causing OS in the CNS. They found that these vesicles alter signaling pathways and activate inflammasomes, potentially resulting in NDs [155]. Ullah and colleagues [156] showed that EVs (metabilites) from the GM may impact OS in the CNS, affecting the progression of NDs, including PD and AD. Furthermore, Wang et al. [157] proposed a relationship between gut health and brain illnesses by arguing that disruption of the GM may increase OS and impair redox homeostasis, therefore contributing to NDs.

Moreover, numerous investigations have demonstrated that intestinal dysbiosis is a condition marked by higher calprotectin levels in the fecal substance of patients with AD [158]. In addition, a large number of bacterial strains in the digestive tract have the ability to control neural system function through the production of neurotransmitters [159]. Decreased levels of specific bacterial taxa, notably *Bifidobacterium*, impact the synthesis of Ach (Acetylcholine) and other neurotransmitters in AD patients. This points to a possible peripheral‐to‐central link between dysbiosis of the gut, dysregulation of neurotransmitters, and OS [160]. Therefore, the OS in NDs is substantially influenced by GMEVs, which presents potential therapeutic opportunities for disease management and neuroprotection.

The relationship between neuro‐inflammation and OS is intricate. Inflammatory cytokines are produced when OS activates nuclear factor‐kappa B (NF‐κB), a critical transcription factor in the inflammatory process. Numerous NDs are associated with increased NADPH oxidases (NOX), an enzyme family that generates ROS as a metabolic byproduct. NOX also plays a role in neuro‐inflammation and OS [161].

GMEVs have been related to the activation of CNS resident immune cells, astrocytes, and microglia by delivering bioactive chemicals such as LPS, lipoproteins, and miRNAs [162]. These EV‐associated molecules have the ability to attach to microglia and astrocyte surface‐expressed pattern recognition receptors, starting intracellular communication cascades that ultimately result in the release of inflammatory cytokines [163, 164]. LPS, components of GMEVs found in the outer membrane of gram‐negative bacteria, are highly active molecules that can strongly activate microglia and astrocytes [164, 165]. When toll‐like receptor 4 (TLR4) detects LPS, microglia and astrocytes undergo a phenotypic transformation that involves changes in their shape and the release of pro‐inflammatory cytokines, including tumor necrosis factor‐alpha (TNF‐α), interleukin‐1 beta (IL‐1β), and interleukin‐6 (IL‐6). These cytokines have a role in attracting and activating immune cells outside the CNS, leading to the spread of inflammation in the brain [164, 166].

Furthermore, Han et al. [28] showed that when GMEVs Of *Aggregatibacter actinomycetemcomitans* were administered intracardially to mice, they successfully bridged the BBB and transferred exRNA cargos from GMEVs to host macrophage cells. Activation of toll‐like receptor 8 and the resulting NF‐κB signaling pathway induced TNF‐α production in the brain via the secretion of short RNAs by OMVs. GMEVs were shown to be responsible for the upregulation of TNF‐α expression, which is believed to contribute to the development of neuroinflammatory conditions such as AD [28]. Another study showed that the periodontopathogen *A. actinomycetemcomitans* releases GMEVs, which may activate NF‐κB and lead to neuro‐inflammation and an increase in IL‐6 levels in the brain by delivering exRNAs to monocytes and microglial cells [128].

In addition, GMEVs may transport lipoproteins that may stimulate microglia and astrocytes by interacting with cell surface receptors such as toll‐like receptor 2 and CD14. In response to lipoprotein detection, inflammatory signaling pathways are activated, leading to the production of chemokines and cytokines that promote neuro‐inflammation [167].

Furthermore, recent studies have highlighted the role of GMEVs‐derived miRNAs in regulating neuroinflammatory responses in the CNS. GMEVs‐associated miRNAs can be taken up by microglia and astrocytes, where they modulate the expression of target genes involved in inflammatory signaling pathways [168]. In addition, microbial metabolites such as tryptophan, butyrate, acetylcholine, norepinephrine, serotonin, and dopamine can indirectly affect miRNA biology by regulating astrocyte function and maintaining the integrity of the BBB. These metabolites may also impact human behavior by disrupting normal levels of neurotransmitters [167, 169].

Hence, the available research findings suggest that GMEVs substantially impact regulating neuro‐inflammation in the CNS by transporting bioactive substances such as LPS, lipoproteins, and miRNAs. The activation of microglia and astrocytes and the increase of pro‐inflammatory cytokines and chemokines lead to neuronal damage and worsen NDs.

### 4.2 Link between GMEVs and protein misfolding and aggregation in NDs

NDs are often identified by the buildup of proteins that have misfolded due to improper post‐translational modification. These proteins include TDP‐43 in ALS, α‐synuclein in PD, and β‐amyloid in AD [168, 170, 171, 172].

Recent findings indicate that GMEVs may play a role in the spread of protein misfolding and aggregation in the CNS, influencing the onset and progression of NDs [172, 173, 174, 175]. A study found that mice over‐expressing αSyn had more αSyn aggregates in their brains than germ‐free mice. Additionally, when germ‐free mice were given specific bacterial metabolites orally, it increased neuro‐inflammation and motor symptoms. These findings suggest that the GM and their small molecules (metabolites), specifically in the form of GMEVs transmission, may play a significant role in αSyn pathology and microglia activation in PD [149]. GMEVS of individuals with PD were shown to cause the aggregation of αSyn, a characteristic feature of PD pathology, in both in vitro and in vivo [124, 176].

Research has shown that certain elements of GMEVs, such as LPS, can move from the blood to the brain, triggering a response in microglia and worsening amyloid aggregation, which in turn plays a role in the advancement of neurodegenerative conditions [177, 178, 179].

In addition, GMEVs may alter autophagy lysosomal processes, which are essential for eliminating clumped or misfolded proteins. Dysregulation of these pathways may speed up the development of a disease. Autophagy lysosomal processes or pathways are a biological mechanism that eliminates misfolded proteins, malfunctioning organelles, and pathogenic bacteria by degrading and reusing them [180, 181].

The autophagy lysosomal process is very important to prevent the accumulation of αSyn in PD, Aβ, and phosphor‐tau in AD and to facilitate the removal of these harmful proteins when triggered in the brain. Furthermore, the irregular control of the autophagy lysosomal process has been associated with the stimulation of inflammatory pathways and OS, both of which have a role in the progression of NDs [182].

Multiple studies have revealed that manipulating the autophagy lysosomal process through GMEVs has shown promise as a potential therapeutic approach for addressing NDs. Specifically, it has been suggested that GMEVs could target the autophagy lysosomal mechanism [125, 130, 132, 176, 183, 184]. Thus, GMEVs have the potential to play a role in the development of NDs. They can impact protein misfolding, neuro‐inflammation, and disruptions in autophagy lysosomal processes, opening up possibilities for new therapeutic approaches.

Although the role of GMEVs in protein aggregation shows promise as a research area, there are still significant hurdles to overcome. The precise composition and biological roles of GMEVs still need to be wholly understood, and further investigation is required to elucidate the processes via which they impact protein aggregation. Moreover, it is essential to create technology that can modify GMEVs in a regulated and exact way to use these discoveries in therapeutic settings effectively.

### 4.3 Link between GMEVs and mitochondrial functions in NDs

Mitochondria have crucial functions in developing and maintaining brain networks, generating energy, facilitating cellular communication, and regulating programmed cell death, known as apoptosis. Mitochondria dysfunction is a characteristic feature of NDs [185]. Studies suggest that GMEVs can influence mitochondrial function via several mechanisms, such as controlling the production, movement, and breakdown of mitochondria, a process called mitophagy [185, 186]. In a recent study, Deo et al. [129] showed that GMEVs from *E. coli* cause mitochondrial malfunction, mitochondrial death, and trigger inflammation in PD.

Esteves et al. [135] tested the hypothesis that harmful compounds produced by the GM increase neuro‐inflammation in PD using a mouse model and cell cultures. They discovered that by reducing intestinal permeability, β‐N‐methylamino‐l‐alanine, which the GM produces, penetrates the brain and passes the BBB. It causes neuronal damage, particularly because of mitochondrial failure [135]. Hence, these data suggest that GMEVs have a significant role in causing mitochondrial dysfunction and neuro‐inflammation, which are believed to be involved in the development of NDs.

MiRNAs and proteins are among the signaling molecules shown to be carried by GMEVs and directly interact with mitochondrial pathways [23, 47]. It has been indicated that GMEVs possess distinct miRNAs that can control mitochondrial genes related to energy metabolism and apoptosis. An imbalance in the regulation of these miRNAs might result in impaired functioning of mitochondria, which in turn contributes to the development of neurodegenerative processes [187]. GMEVs may transport proteins involved in mitochondrial dynamics, including those that regulate mitochondrial fission and fusion. The maintenance of mitochondrial integrity and function depends on these dynamics, and their disturbance has been linked to the development of NDs [188].

### 4.4 Link between GMEVs and BBB dysfunction in NDs

**Figure 4:**
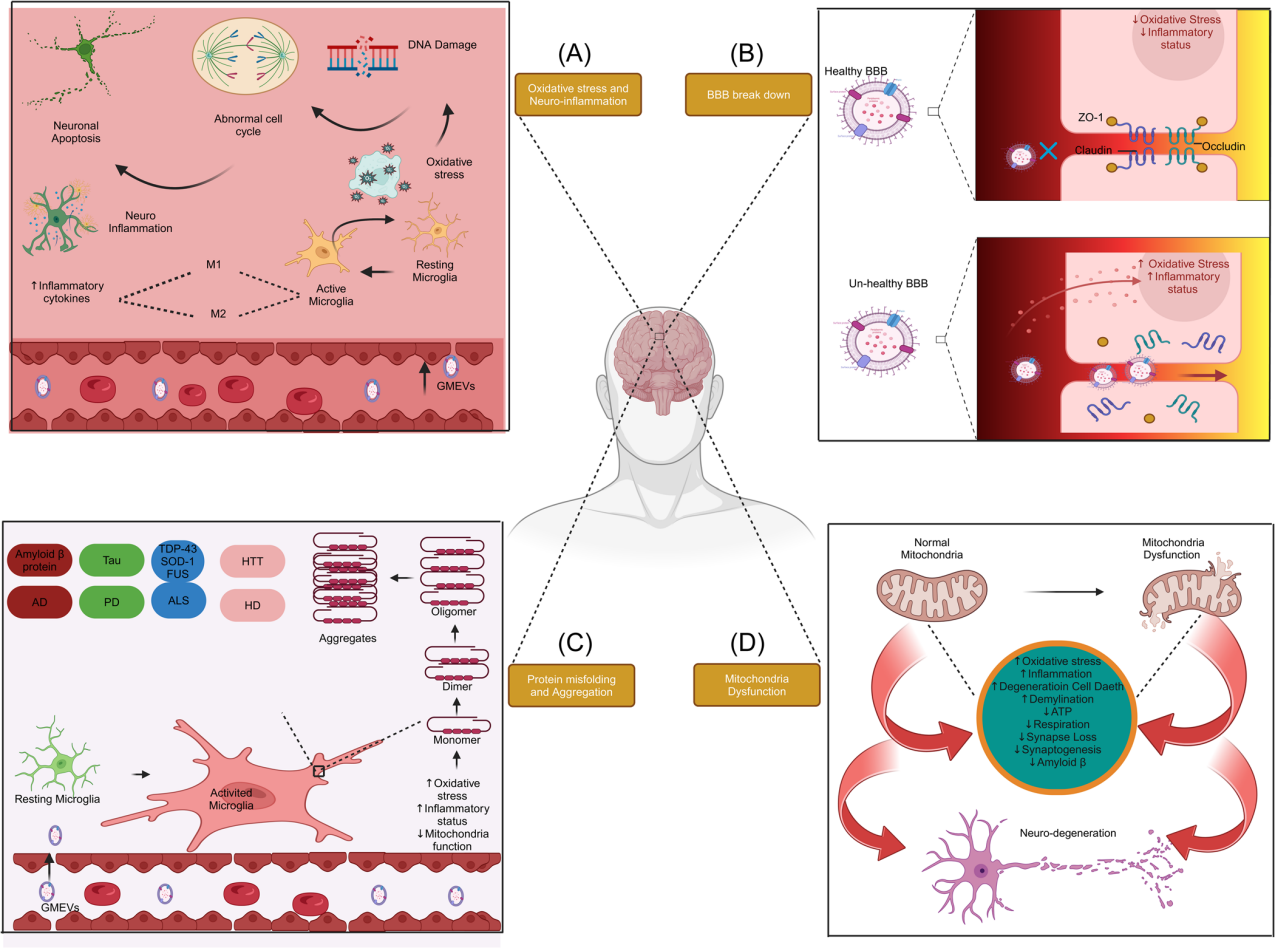
Possible pathway underlying gut microbiota extracellular vesicles on neurodegenerative disorders. (A) Oxidative stress and neuro‐inflammation, (B) blood‐brain barrier breakdown, (C) protein misfolding and aggregation, (D) Mitochondria dysfunction. *Note*: ↑ means increase, ↓ means decrease.

The BBB is essential for controlling the movement of molecules between the circulation and the brain parenchyma, which is necessary for the brain's metabolic processes and neuronal function. Hence, it is essential to protect the functional and structural integrity of the BBB to uphold the equilibrium and stability of the brain's microenvironment [189, 190]. Various NDs are accompanied by disruption of the BBB, leading to neuro‐inflammation, neuronal dysfunction, and neurodegeneration [191, 192]. Research has shown that GMEVs play a significant role in influencing the integrity and function of the BBB [149]. It has been demonstrated that GMEVs can traverse this barrier, enhancing its permeability via inflammatory or infectious mechanisms. There have been studies indicating the existence of bacterial nucleic acids in the brains of deceased people who had AD. The genetic material may have entered GMEVs by exploiting the compromised BBB in individuals with AD [95]. This might result in GMEVs or pathogens, such as bacteria, crossing the BBB and establishing themselves in the brain. Recent research has shown that GMEVs can directly penetrate the BBB [28]. Multiple studies have demonstrated that GMEVs containing LPS may promote vascular permeability and BBB failure by inducing endothelial cell death and disrupting tight junction proteins [141, 193, 194]. The translocation of LPS into the bloodstream and the brain can occur when the gut barrier and BBB are compromised. This can lead to the activation of TLR4 on microglia, which worsens the aggregation of amyloid and the progression of NDs [177, 178, 179]. The findings unequivocally demonstrate that GMEVs play a role in compromising the integrity of the BBB and contributing to the advancement of NDs. Following pathways discussed above are shown in Figure 4.

## 5 EXPERIMENTAL APPROACHES AND METHODOLOGIES

To gain a deeper understanding of the relationship between GMEVs and NDs, it is crucial to have sensitive, accurate, and standardized bioassays to determine the composition and molecular profile of EVs in clinical samples. This is essential for the quick clinical application of EVs [195]. Due to their distinct size characteristics, EVs are intriguing subjects that stand apart from conventional analysis targets. They possess a size smaller than cells yet larger than proteins [196, 197]. Isolation techniques are essential for obtaining pure GMEVs suitable for downstream analyzes [198]. Ultracentrifugation, a well‐established technique, utilizes a series of centrifugation steps at varying speeds to separate GMEVs according to their size and density [54, 88, 199]. Similarly, density gradient ultracentrifugation and size exclusion chromatography offer alternatives for GMEVs isolation, ensuring superior purity and maintaining the integrity of GMEVs [200, 201, 202, 203, 204]. Recently, Wei et al. [205] introduced a new method for isolating GMEVs using an antimicrobial substance called epsilon‐poly‐l‐lysine (ϵ‐PL). This method allows for the precipitation of GMEVs at a lower centrifugal speed of 10,000*g*, offering an alternative to the standard ultracentrifugation method [205].

However, despite their effectiveness, these methods have limitations, as exhibited in Table 2. Performing these experiments can be quite time‐consuming and often necessitates specialized equipment. Additionally, there is a risk of co‐isolating non‐GMEVs contaminants [206], which can compromise the purity of the GMEVs preparations. To overcome these limitations, scientists are investigating new methods of isolation, including techniques based on microfluidics. These approaches aim to enhance the isolation process's efficiency and purity.

**Table 2:** Effectiveness and limitations of the isolation methods.

Technique	Duration	Effectiveness	Limitations	Reference
Ultra‐centrifugation	140–600 min differential centrifugation: 300*g*, 10,000*g*, 100,000–200,000*g* (1.5 h)	**Financial Considerations:** Examining ultracentrifugation costs. **Sample isolation efficiency:** Measures ultracentrifugation's ability to isolate samples from large volumes to maximize yields. **To maintain chemical purity:** Samples are ultra‐centrifuged without adding chemicals to maintain chemical purity and create a controlled environment for further tests.	**Apparatus and Operations:** Ultracentrifugation requires specialist equipment and is complicated, thus careful attention is needed. **Non‐Exosomal Impurities:** These can contaminate downstream analyzes and impact exosome purity Ultracentrifugation may be unreliable in research applications due to limited reproducibility. **Low RNA yield:** The method generally yields low RNA, which might make molecular analysis difficult. **Rotor efficiency and exosome integrity:** It can be affected during ultracentrifugation, with factors like rotor type and centrifugal force affecting efficiency. **Sample processing capacity:** The sample processing capacity is limited to six samples at once, which may limit throughput for large‐scale studies or clinical applications.	[195, 207, 208, 209]
Density gradient ultra‐centrifugation	250 min–2 days	**Devoid of Contamination Results:** Obtaining samples with little contaminants remaining. **Purity of preparation:** This method successfully separates the desired viral particles while reducing contamination with nontarget components. Absence of additional ingredients.	**Complexity:** It requires specialized protocols and technical skills for lab application. **Loss of sample:** High sample loss during ultracentrifugation can damage experimental results and impair yield. Fails to distinguish big vesicles with identical sedimentation rates.	[210, 211, 212]
Size‐exclusive chromatography	1 mL/min + column washing (Polymer‐filled columns with varying pore sizes)	Preserves the integrity of GMEVs by maintaining their native form and function. Achieves high detection and restoration by smoothly isolating GMEVs with minimal loss. Adapt to multiple sample quantities, making it a scalable process. Generates a substantial amount of GMEVs for downstream analysis, ensuring high GMEV yield. Maintains GMEVs stability throughout the separation process, preventing aggregation. Effectively handles samples of various viscosities, regardless of their thickness. Frequently provides excellent EV preparations with low contamination, ensuring excellent reproducibility and purity. Effectively eliminates non‐GMEVs contaminants using high ionic strength buffers, resulting in effective elimination of impurities. Simplifies the isolation process by avoiding the use of extra substances, making it chemical‐free.	**Peak resolution:** Peak resolution is restricted and achieving effective separation necessitates a significant disparity in molecular weight (≥10%) between the different components. **Specialized equipment:** Requires specific chromatographic equipment, which may be expensive and difficult to acquire. **Complex procedure:** Involves a sequence of specific instructions and specialized expertize. **Co‐isolation:** Concerns about potential contamination arise from the identification and isolation of protein aggregates and lipoproteins, as they have the potential to affect the purity of extracellular vesicles. **Efficiency:** Efficiency is limited as it can only handle a single specimen at a time. **Coast:** Administrative costs may be quite high, as both the tools and materials often come with a considerable cost.	[213, 214]
Microfluidic technologies	1–14 µL/min	**Efficiency:** Allows for rapid handling of specimens. **Purity:** Exceptionally clean EV preparations are delivered. **Outstanding performance:** Maintains GMEVs bioactivity and functionality at a high level.	**Tool level of complexity:** The need for intricate microfluidic designs and careful production. **Additional equipment:** Requires particular tools for function and assessment. **Costly:** Developing, manufacturing, and operating can be quite expensive.	[215, 216]

Once isolated, analyzing the cargo of GMEVs becomes crucial for gaining insight into their functional roles. Proteomics [202], RNA sequencing, and lipidomics are frequently used to analyze EVs cargo components [217, 218]. Using mass spectrometry‐based proteomics, scientists have comprehensively understood the proteins found in EVs. This has provided valuable insights into these proteins' roles in NDs [219]. RNA sequencing enables the discovery of RNA species associated with EVs, such as mRNAs and miRNAs, offering valuable insights into their regulatory roles [220]. In addition, lipid profiling techniques provide insights into the lipid composition of EVs membranes and their role in signaling pathways [221].

Although cargo analysis provides a wealth of information, omics approaches still present particular challenges. Specialized expertize is necessary for these tasks, as they deal with technical variability and potential scarcity of specific cargo molecules. Therefore, it is crucial to validate findings using different methods and integrate various omics approaches to ensure the trustworthiness of results.

Functional assays enhance cargo analysis by offering a deeper understanding of the biological impacts of GMEVs. Cell culture models [207], using neuronal or glial cell lines, enable researchers to study the impact of EVs on cellular processes related to neurodegeneration [208]. Animal models offer valuable insights into the relevance of in vivo conditions, allowing for the evaluation of the impact of EVs on the progression of diseases and neuropathology [209]. By utilizing biochemical assays, scientists can measure GMEVs cargo molecules' specific activities or interactions. This approach helps to gain a better understanding of the functional implications of GMEVs cargo [207, 210].

Nevertheless, making sense of findings from functional assays can prove quite tricky, given the diverse nature of GMEVs and the intricate nature of NDs. Incorporating different methods, like organoid cultures or co‐culture systems [211] can improve the accuracy and applicability of research findings.

Ultimately, a comprehensive approach that combines various techniques and assays is crucial for understanding the impact of GMEVs on NDs. Further progress in technology, protocol standardization, and validation of research findings will drive advancements in this field, resulting in a greater comprehension of disease mechanisms and potential therapeutic interventions.

## 6 THERAPEUTIC PROSPECTS AND PRACTICAL IMPLICATIONS OF GMEVS

The investigation into the role of GMEVs in NDs offers exciting potential for clinical applications. These nanovesicles produced by GM generated interest in their possible role in the gut‐brain connection, impacting neurological function and the progression of diseases.

### 6.1 GMEVs as diagnostic biomarkers

One important aspect is their evaluation as diagnostic biomarkers [212]. Research has indicated changes in the composition of GM in different NDs, including AD and PD [222]. In a study conducted by Park et al. [223], they proposed using blood sample‐associated GMEVs as a potential method to detect GM dysbiosis in individuals with NDs.

GMEVs, which carry microbial cargo, might be used as markers of dysbiosis or the presence of illness, helping with the early detection or tracking of disease development. For example, the GMEVs released by *A. actinomycetemcomitans* can enter the mouse brain and may contribute to neuroinflammatory disorders by carrying exogenous RNA cargo [28].

### 6.2 GMEVs as therapeutic agents

In addition, the intriguing future promise of GMEVs has captured attention as they pursue innovative treatment methods. GMEVs have been studied as possible therapeutic targets to adjust the composition of the GM to alleviate neurodegenerative processes. In animal models of AD, the immune system is significantly impacted by GMEVs derived from specific strains of GM, including *Bifidobacterium bifidum* [125, 126]. The administration of these GMEVs led to a significant decrease in brain inflammation and a notable enhancement in overall brain function. These intriguing findings indicate that GMEVs have noteworthy protective effects on neurons [125].

Moreover, studies have found promising applications for GMEVs such as *Akkermansia muciniphila*. In a mouse model of PD, these GMEVs showed a remarkable ability to decrease neuro‐inflammation [133, 134]. This effect is attributed to their ability to lower the levels of pro‐inflammatory cytokines in the brain and increase the presence of anti‐inflammatory cytokines [130, 176]. These findings emphasize the ability of GMEVs to regulate the immune response in NDs, indicating a promising area of study and the possible use of GMEVs as an effective therapeutic method for treating these debilitating ailments [136, 183].

### 6.3 GMEVs as drug delivery systems

Concurrently, initiatives at translational research and ongoing clinical studies are vigorously investigating the use of GMEVs in disease treatment. Among the many areas these initiatives cover is advancing the development of EV‐based drug systems for delivery. Researchers intend to create EVs to target particular brain areas or deliver therapeutic compounds, including neuroprotective drugs or gene treatments, using their inherent capacity to overcome biological barriers and carry cargo [224]. In addition, GMEVs have the ability to transport medicinal drugs along with their associated enzymes, which may help them interface with and penetrate host epithelial cells. Due to their capacity to cross the BBB, GMEVs offer tremendous promise. This makes them a promising candidate for drug delivery systems because BBB is a common barrier to delivering drugs to the brain [111, 225, 226, 227, 228]. Promising outcomes have been shown using BEVs utilized as drug delivery agents in model organisms. Given that, GMEVs were permitted to transport therapeutic agents like small interfering RNA (siRNA) and drugs to specific brain cells [212, 229]. For instance, EVs derived from mesenchymal stem cells delivered active siRNA targeting the tau protein. As a result, Tau phosphorylation and aggregation were diminished in a murine model of AD [229, 230]. In a recent study, Chen et al. [231] conducted experiments using a combination of nano‐gold and GMEVs to improve the effectiveness of radiotherapy and immunotherapy against glioblastoma. The results showed promising outcomes, including an extended survival period for mice [231]. Furthermore, GMEVs have the potential to be internalized by nearby neutrophils, triggering a response in microglia. This suggests that GMEVs could serve as a means to transport drugs specifically to microglia [213].

### 6.4 GMEVs as therapeutic vaccines

**Figure 5:**
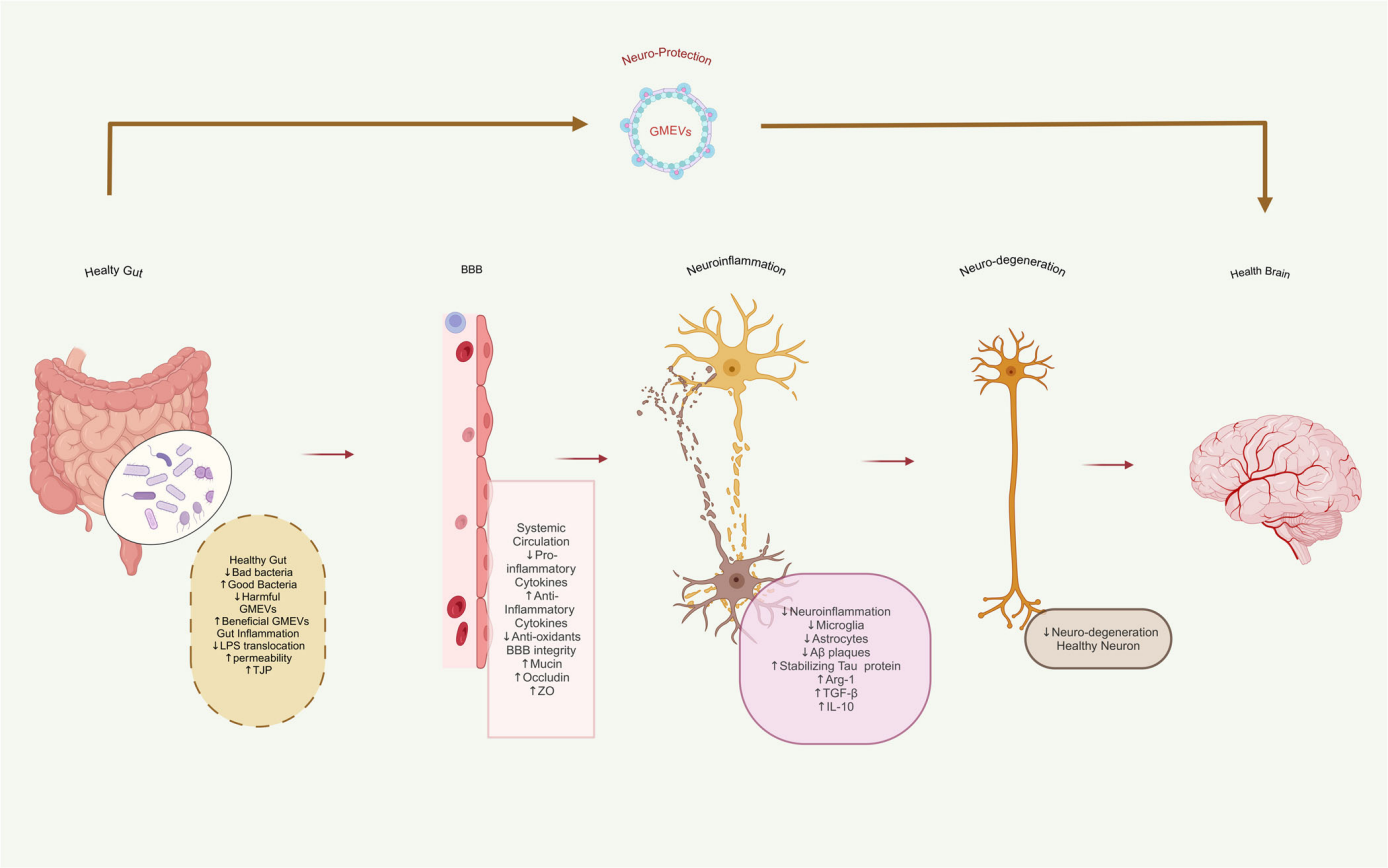
Therapeutic application of GMEVs. Gut microbiota extracellular vesicles could positively impact different aspects of the gut‐brain axis, leading to improved brain health. Balance gut microbiome, improve the blood‐brain barrier integrity, decrease neurological inflammation, and finally, hinder neurodegeneration. *Note*: ↑ means increase, ↓ means decrease. GMEV, gut microbiota extracellular vesicle.

As mentioned above, gut dysbiosis is the leading cause of NDs. Therefore, using GMEVs as therapeutic vaccines is a good strategy. These vaccines prevent infectious agents from producing damaging chemicals instead of directly attacking the germs. This aids in preventing microbial alterations that may result in illnesses [214]. For instance, a vaccination against common microbial sugars elicited a potent immune response derived from *E. coli* Nissle 1917 (EcN) GMEVs. Compared to conventional vaccines, which are often costly and labor‐intensive, this one worked better [215]. The therapeutic application of GMEVs is shown in Figure 5.

### 6.5 Pros and cons of GMEVs

GMEVs provide many health and disease management benefits. These vesicles can influence host immune responses to increase tolerance or reduce inflammation [19, 47, 216, 233]. GMEVs are advantageous for medication administration because of their unique composition and biocompatibility. GMEVs are intriguing candidates for vaccination because they can enhance immune responses and antigen presentation. They also assist in the identification of diseases because their load may reveal the host's pathological condition [19, 216].

Investigating and utilizing GMEVs may have certain advantages, yet there is also some important cons. primarily compared to exosome characterization, GMEV characterization is still in its early phases due to the complexity and diversity of gut bacterial populations. Further optimization of the processes for isolating and purifying GMEVs is required to achieve uniformity and reproducibility. Furthermore, it is yet unclear how GMEVs act in different pathogenic situations, prompting further study to close this information gap. The fact that various bacterial species or strains may alter the composition and functionality of GMEVs further complicates the findings. These obstacles must be overcome to ensure the effective use of GMEVs in biomedical research and to fully utilize them as treatments in clinical applications [19, 47, 232]. We provided the GMEVs pros and cons, which are shown in Table 3.

**Table 3:** Benefits and key difficulties of bacteria extracellular vesicles (BEVs).

	Easy industrialization	Drug delivery	Modifiability	Diagnostic tool	Reference
Benefits	Bacteria have commercial benefits over eukaryotes. Fast growth, high‐density cultures, and gene editing are benefits. Fermentation by bacteria is cheap and scalable. Industrial uses for BEVs are promising. E.g.: Anti‐osteoporotic probiotic A. muciniphila patent opens industrial use.	Nano‐sized BEVs increase medication absorption. Their stability prevents medication deterioration. For instance, BEVs deliver Alzheimer's RNA.	BEVs load molecules in vivo or in vitro. Cells are treated to create BEVs in vivo. Electroporation or genetic engineering is used in vitro. E.g.: Electroporation loads siRNA and nanoparticles.	BEVs diagnose infection. They spread with bodily fluid‐borne bacteria. Their capacity to stimulate immunological responses make them promising vaccine candidates.	[95, 129, 130, 131, 233, 234]
	**Uncertain mechanism**	**Biological safety**	**Time consuming**	**Diverse contents**	
Key difficulties	Not sure how BEVs penetrate cells. Unknown mechanisms and interactions. Uncertain BEV component packaging.	Unsafe BEVs may include LPS. Immune responses may occur with LPS. Solution: use LPS‐free bacteria or neutralize LPS.	Poor BEVs isolation techniques. Require faster, cheaper methods. BEVs are hard to distinguish.	BEVs have diverse molecules. Bacteria and conditions affect composition. Investigation is needed on the packaging mechanism.	[129, 130, 163, 164, 233, 235, 236, 237, 238, 239, 240]

Abbreviations: BEV, bacteria extracellular vesicles, LPS, lipopolysaccharide; siRNA, small interfering RNA.

GMEVs offer a hopeful solution for neurological disorders, delivering a wide range of beneficial substances like lipids, small molecules, and miRNAs. Through this release, GMEVs have a complex impact, coordinating different mechanisms to improve neurological well‐being. GMEVs support neuronal function, balance microglial activity, promote BBB integrity, reduce inflammation and OS, and stabilize intestinal integrity. This all‐encompassing effort emphasizes how GMEVs may be a viable therapeutic approach for neurological diseases.

## 7 CONCLUSION AND FUTURE PROSPECTUS

GMEVs, which have a diverse role in the etiology of NDs, impact OS, neuro‐inflammation, mitochondrial dysfunction, and other cellular processes. It is crucial to comprehend the mechanisms underlying these effects and the specific cargo molecules involved to develop novel methods for treating NDs by targeting the GBA. Lately, a significant amount of interest has been drawn to research efforts that investigate the function of GMEVs in NDs.

On the one hand, there is a fascinating narrative regarding the potential involvement of GMEVs in developing NDs. The GMEVs is a type of vesicle of critical importance. Because of their astonishing capacity to influence a variety of processes, including neurological inflammation, OS, protein aggregation, and malfunctioning mitochondria, and their capacity to penetrate the BBB to reach the brain, it is possible that they play an essential part in the development of NDs. In contrast, GMEVs may 1 day be used as a treatment for NDs because of their unique characteristics. Their remarkable capacity to impact immune responses and transcend biological barriers makes them particularly appealing to targeted drug delivery and immunomodulation in various therapeutic contexts. The ability of GMEVs to traverse the BBB and induce defense mechanisms towards antigens is an important outcome that could lead to their application in multiple therapies for neurological disorders.

There is hope for the future of vaccine development in the form of GMEVs produced from probiotics, which can elicit robust immune system responses. Their ability to accomplish this demonstrates their potential for facilitating targeted drug delivery to the brain, restoring a healthy balance in the microbiota, and enhancing the immune system's defense against pathogenic bacteria in the digestive tract. GMEVs vaccination can alleviate neuro‐inflammation and ward against NDs. Also, they could help us learn more about the causes of NDs, which could lead to the development of new treatments.

There are a number of considerations that must be made while studying and choosing GMEVs for NDs. Disparate data obtained from different approaches in GMEVs separation and characterization is a major difficulty. It is difficult to make meaningful comparisons among studies due to the inconsistent results caused by the lack of standardization in the methods used.

More research is required to fully understand the intricate relationships between probiotics and neurological disorders. The specific ways in which GMEVs impact neuroprotection and cognitive performance remain unclear in the existing evidence. Hence, well‐designed clinical trials are desperately needed to evaluate the effects across various age groups. These trials should use standardized extraction and characterization techniques and include diverse populations.

Ultrafiltration, density gradient centrifugation, and size exclusion chromatography are three methods that have recently been detailed for separating GMEVs from human bodily fluids. Another challenge is the current lack of a reliable way to determine the mother bacterial origin of GMEVs or their content in microbial communities that are diverse and heterogeneous, like the GM. Future study is needed to show how the variability of GMEVs contents and production connects with the variability of the parent microbiome. Moreover, further investigation is required to evaluate the following aspects of GMEVs packaging:

- The reasons behind the molecular packing.
- The targeting of particular cells.
- How cargoes are released.
- GMEVs can traverse biological barriers like the blood‐brain and intestinal barriers.

## AUTHOR CONTRIBUTIONS

**Adil Hassan**: wrote the manuscript. **Mahjabina and Otmilda Hapsari Sudiro**: made the figures. **Guixue Wang and Xiaoling Liao**: supervised this project. **All other authors**: helped in revising the final manuscript and approved it for publication.

## CONFLICT OF INTEREST STATEMENT

The authors declare no conflicts of interest.

## ETHICS STATEMENT

No animals or humans were involved in this study.

## Data Availability

No new data and scripts were generated in this review. Supplementary materials (graphical abstract, slides, videos, Chinese translated version and update materials) may be found in the online DOI or iMeta Science http://www.imeta.science/imetaomics/.
